# Identifying markers of cachexia and nutritional deficit on radical radiotherapy outcomes in lung and head and neck cancer

**DOI:** 10.1097/SPC.0000000000000818

**Published:** 2026-07-08

**Authors:** T.K. Liu, D. Noble, M. Stares, I. Phillips

**Affiliations:** a University of Edinburgh Medical School, University of Edinburgh, Edinburgh, Scotland; b Edinburgh Cancer Centre, Western General Hospital, Edinburgh, Scotland; c CRUK Scotland Centre, Institute of Genetics & Cancer, University of Edinburgh, Edinburgh, Scotland

**Keywords:** cancer cachexia, head and neck cancer, lung cancer, radiotherapy, systemic inflammation

## Abstract

**Purpose of review:**

Cancer cachexia is a multifactorial syndrome characterised by weight loss, muscle wasting, malnutrition, and systemic inflammation. Although well-recognised in advanced cancer, its significance in patients receiving radical radiotherapy for potentially curable malignancies is less well understood. This review examines the impact of cachexia-related biomarkers in patients undergoing radical radiotherapy for lung or head and neck cancers.

**Recent findings:**

Many studies evaluating cachexia-related phenotypes do not explicitly use the term ‘cachexia’. Therefore, this review focuses on four key domains of cachexia: weight loss, malnutrition, sarcopenia, and systemic inflammation. Across both tumour types, these biomarkers were frequently associated with poorer survival, increased treatment toxicity, and reduced treatment tolerance in patients receiving radical radiotherapy with curative intent for lung or head and neck cancers. However, the literature is dominated by retrospective studies employing heterogeneous definitions in poorly defined patient groups.

**Summary:**

Cachexia-related biomarkers provide important prognostic information in patients receiving radical radiotherapy for lung cancer and head and neck cancer. Routine assessment of weight loss, nutritional status, body composition, and systemic inflammation may improve risk stratification and support personalised treatment decisions. Future prospective studies should incorporate standardised cachexia assessments to validate their clinical utility and evaluate emerging nutritional, prehabilitation, and anti-cachexia interventions.

## INTRODUCTION

Cachexia is a complex metabolic condition characterised by unintentional weight loss, muscle loss, fat depletion, and systemic inflammation that cannot be reversed by conventional nutritional support alone [1▪]. Cancer cachexia arises from complex tumour–host interactions that drive metabolic imbalance through inflammatory cytokine release, altered energy homeostasis, and the suppression of anabolic pathways [2,3]. Its clinical consequences extend beyond nutritional impairment, contributing to reduced physical function, diminished treatment tolerance, and inferior survival across cancer types and treatment settings [4–6].

The definition of cachexia has evolved. Fearon *et al.* provided an initial consensus definition with explicit diagnostic thresholds, defining cachexia as weight loss exceeding 5% over 6 months, or weight loss exceeding 2% in individuals with a body mass index (BMI) <20 kg/m^2^ and/or established sarcopenia [1▪]. Sarcopenia itself is defined as the progressive loss of skeletal muscle mass and function and is commonly associated with adverse health outcomes [7]. More recently, the Global Leadership Initiative on Malnutrition (GLIM) established consensus criteria for the diagnosis of malnutrition, requiring at least one phenotypic criterion (unintentional weight loss, low BMI, or sarcopenia) and at least one aetiological criterion (reduced food intake/assimilation or disease burden/inflammation). These criteria can be applied to diagnose cancer cachexia when combined with a measure of systemic inflammation (e.g., C-reactive protein [CRP]), as cachexia is increasingly recognised as a syndrome of disease-related malnutrition driven by systemic inflammation [8,9].

**KEY POINTS**

- Cachexia is common condition in lung and head and neck cancer patients receiving radical radiotherapy and is associated with poor outcomes.
- Weight loss, malnutrition, sarcopenia, and systemic inflammation each show prognostic value in patients receiving radiotherapy.
- Current evidence is largely retrospective, with heterogeneous definitions limiting routine clinical implementation.
- Prospective trials should include standardised cachexia assessments to validate biomarkers and guide future interventions.

The prognostic significance of cachexia is well established in patients receiving systemic anticancer therapies, where weight loss, malnutrition, sarcopenia, and inflammatory biomarkers predict treatment toxicity, lower quality of life, and reduced survival [10–16]. However, less is known about the impact of cachexia in patients receiving radical radiotherapy (RRT), with or without chemotherapy. A recent systematic review identified only 9 out of 57 cachexia clinical trials reporting oncological endpoints that included radiotherapy as a treatment for all patients, with a further 16 studies including it for at least some patients [17].

RRT ± chemotherapy is the standard-of-care treatment option for patients with inoperable locally advanced lung or head and neck cancers (HNC). These tumour groups were selected for review because they share several characteristics associated with cancer cachexia, including a substantial comorbidity burden, significant treatment-related toxicity, and a high prevalence of nutritional impairment and systemic inflammation [18,19]. The evaluation of cachexia-related biomarkers in patients with HNC and lung cancer receiving RRT also deserves particular consideration. Unlike many other cancer populations, such patients frequently experience nutritional compromise directly attributable to tumour- and/or treatment-related symptoms. Consequently, cachexia and symptom-driven nutritional decline frequently coexist, making it difficult to distinguish between reversible nutritional deficits and the irreversible metabolic disturbances that characterise established cachexia.

We aimed to identify research using one of four different biomarkers of cachexia: ‘weight loss’, ‘malnutrition’, ‘sarcopenia’, or ‘systemic inflammation’. Significant weight loss is commonly defined as >5% of weight in 3–6 months. Malnutrition can be assessed by % measured weight loss or with validated screening tools such as the Malnutrition Universal Scoring Tool or Patient-Generated Subjective Global Assessment (PG-SGA). Systemic inflammation can be evaluated using routinely available blood tests, including albumin, CRP, lactate dehydrogenase, white cell count, neutrophil count, and/or lymphocyte count, either individually or incorporated into a composite prognostic score [20].

This methodology was chosen as studies frequently evaluate only one aspect of the four biomarkers of cachexia and, anecdotally, do not explicitly use the term ‘cachexia’. This review highlights site-specific findings, methodological limitations, and key areas for future research.

## HEAD AND NECK CANCER

Understanding the impact of both sarcopenia specifically and cachexia more broadly is particularly important in patients undergoing RRT for HNC. The nature of the disease and its treatment can lead to mechanical and symptomatic impediments to eating that represent an entirely different phenotype from the cachexic syndrome. Interestingly, cachexia is increasingly recognised as a major prognostic factor in patients with HNC undergoing RRT, and cachexia-related biomarkers can adversely affect treatment tolerance and survival [21▪,^22^].

Sarcopenia has been extensively investigated in HNC. In a meta-analysis of 63 studies involving 14,804 patients, van Heusden et al. showed that radiologically defined sarcopenia was significantly associated with poorer survival (logOR = 0.808, *P* < 0.001), increased treatment-related complications (logOR = 0.669, *P* < 0.001), and greater chemotherapy dose-limiting toxicity (logOR = 0.760, *P* < 0.001) [21▪]. Importantly, treatment modality did not significantly moderate these associations, suggesting a consistent adverse prognostic impact across surgery, chemoradiotherapy, and RRT alone [21▪]. However, heterogeneous cohorts were pooled, radiotherapy-only subgroups were not analysed separately, and there was variability in the detection of sarcopenia, including differences in diagnostic thresholds and anatomical muscle measurements (C3 vs. L3 paraspinal musculature). Furthermore, 90% of studies were retrospective, and several were considered at high risk of bias.

More treatment-specific evidence was reported by Mascarella et al., who analysed 16 observational studies involving 3187 patients, finding that patients undergoing chemoradiotherapy who had sarcopenia were at increased risk of severe short-term toxicity (CTCAE ≥ grade 3; OR = 2.24), comparable to surgical cohorts (odds ratio [OR] = 2.03) [22]. However, no RTT-alone subgroup was analysed, most patients (76%) had stage III–IVA disease, definitions of sarcopenia varied, and the pooled OR for treatment tolerance was not statistically significant (OR = 1.40, 95% confidence interval [CI] = 0.84–2.32), likely reflecting small study numbers and treatment selection bias. Similarly, Vickers et al. reviewed 26 studies of definitive (chemo-)radiotherapy, including seven HNC cohorts. Within HNC populations, sarcopenia independently predicted survival in only 3 of 16 survival outcomes [23]. These inconsistent findings reflect relatively small studies (mean *n* = 227 patients), heterogeneous definitions, and inconsistent adjustment for confounders.

This evidence converges on sarcopenia as an adverse prognostic marker in RRT-based treatment for HNC. However, interpretation is limited by its predominantly retrospective collection basis, inconsistent diagnostic criteria, and frequent combination of RRT and chemoradiotherapy cohorts. Given that patients selected for chemoradiotherapy are typically fitter and biologically distinct from those receiving RRT alone, the independent impact of sarcopenia in patients treated with RRT remains uncertain.

Systemic inflammation may also provide prognostic information for patients receiving RRT for HNC. Koca et al. evaluated nine inflammatory biomarkers in 101 laryngeal SCC patients receiving RRT. On multivariable analysis, an elevated neutrophil-to-lymphocyte ratio (NLR >2.61) independently predicted poorer OS (hazard ratio [HR] = 6.75, 95% CI = 1.86–24.4, *P* = 0.004), whilst CRP predicted inferior progression-free survival (PFS) (HR = 5.03, 95% CI = 1.81–13.9, *P* = 0.002) [24]. Although this study assessed a comprehensive panel of inflammatory biomarkers, its modest sample size, wide CIs, and single-centre retrospective design limit generalisability. Prospective multi-centre studies are required to establish robust prognostic thresholds and determine whether inflammatory biomarkers of cachexia should be incorporated into routine management algorithms.

There is relatively limited recent evidence regarding the impact of malnutrition on HNC patient outcomes. Vílchez-López et al. conducted a multicentre observational study across 12 Spanish hospitals, evaluating 514 HNC patients receiving radiotherapy and finding a malnutrition prevalence of 51.6% by GLIM criteria [25]. Severely malnourished patients experienced significantly poorer OS compared with both moderately malnourished (*P* = 0.010) and well-nourished groups (*P* < 0.001). However, the cohort included all tumour stages (I–IVC), and approximately 60% received concurrent chemotherapy, making it directly relevant only to those patients receiving optimal treatment for locally advanced disease. Nutritional assessments were performed during the first two weeks of treatment, with early treatment-induced deterioration potentially confounding baseline measurements.

In terms of overall weight loss, Toapichattrakul et al. retrospectively analysed 465 HNC RT-receiving patients and found that pre-treatment weight loss exceeding 5% was an independent predictor of prolonged overall treatment time (OTT >49 days; OR = 1.26, *P* = 0.036), alongside concurrent chemotherapy, sarcopenia, and elevated NLR. Notably, 62% of patients experienced prolonged OTT [26]. This is clinically significant because prolonged OTT compromises locoregional control and survival [27]. However, the single-centre, retrospective design, inclusion of both nasopharyngeal cancer and non-nasopharyngeal subtypes, and potential unmeasured confounders (comorbidities, socioeconomic factors) limit generalisability. Importantly, this is one of the few studies that examine multiple elements of cachexia simultaneously. Pre-treatment weight loss is less likely to be reversible in the presence of other factors such as systemic inflammation, highlighting the challenge of disentangling modifiable nutritional deficits from established cachexia. Consequently, strategies such as early intensive nutritional interventions alone may be insufficient, and future strategies may still require specific anti-cachexia approaches, such as anti-cachexia drug therapies.

## LUNG CANCER

Approximately 40% of patients with non-small cell lung cancer (NSCLC) have cancer cachexia [28]. Although a substantial body of literature has examined cachexia biomarkers, much of this evidence is derived from patients with advanced or metastatic disease receiving systemic anticancer therapy. Comparatively, little research has focused on patients receiving RRT, despite it being a recognised treatment option for patients with localised or locally advanced disease [29,30]. RRT technology has rapidly evolved, leading to improved precision and less normal tissue toxicity [31]. However, patients treated with RRT alone tend to be older, more comorbid, and less fit than those selected for surgery or chemoradiotherapy, making the identification of prognostic biomarkers particularly relevant.

Weight loss is a recognised biomarker of poor outcomes in lung cancer [32]. In a meta-analysis of 16 studies involving 6225 patients, Bonomi et al. demonstrated that cachexia, defined as >5% weight loss, was associated with an increased mortality risk (pooled HR = 1.82, 95% CI = 1.47–2.25) [33]. However, only 14.7% of patients received RRT, limiting the applicability of these findings to the RRT population. Alvarez et al. addressed this gap more directly in a single-institution retrospective cohort of 1330 patients receiving radiotherapy for NSCLC. Pre-treatment cachexia, according to Fearon et al.’s definition [1▪], independently predicted inferior survival on multivariate analysis (HR = 1.31, 95% CI = 1.14–1.52, *P* = 0.0002) in all comers, with a particularly marked effect in stage III disease (median survival 15.0 vs. 23.9 months, *P* = 0.009) [34].

Evidence supporting the prognostic significance of sarcopenia in patients receiving RRT for lung cancer is less consistent. Cireli et al. retrospectively studied 54 stage-III patients with NSCLC receiving definitive concurrent chemoradiotherapy and found that 92% were sarcopenic by international L3 Skeletal Mass Index (SMI) thresholds. Neither SMI nor the psoas muscle index predicted survival, whereas low visceral fat tissue area (<37 cm^2^) independently predicted poorer outcomes (HR = 3.8, 95% CI = 1.1–12.7, *P* = 0.032), suggesting that fat reserves, rather than muscle mass alone, may drive prognosis in this population [35]. Interpretation, however, is limited by the small sample size (*n* = 54) and the near-universal prevalence of sarcopenia, which effectively eliminated a meaningful comparator group. In contrast, Zhang *et al*. reported a large prospective multicenter cohort study involving 1881 lung cancer patients and demonstrated that low fat-free mass index independently predicted mortality (HR = 1.29, 95% CI = 1.12–1.49, *P* < 0.001) [36]. However, the association was confined to advanced disease, and no RRT-specific analyses were performed.

Taken together, these findings suggest that abnormal body composition is common amongst patients receiving radical therapy for lung cancer, but the independent prognostic value of sarcopenia remains uncertain. It may be that broader measures of body composition, including visceral adiposity and myosteatosis, provide greater prognostic information than muscle mass alone in this setting. Evidence from our centre suggests that low muscle attenuation, indicating myosteatosis, correlates with poor outcomes in patients receiving chemo-radiotherapy for NSCLC. These observations support a more comprehensive approach to body composition assessment when evaluating cachexia-related risk [37].

Numerous studies have demonstrated associations between biomarkers of systemic inflammation and survival in lung cancer, but few studies have focused specifically on patients receiving RRT. Gomez-Rosas et al. reinforced the prognostic significance of inflammation in a prospective multicenter cohort including 704 patients with advanced NSCLC, demonstrating that baseline high-sensitivity CRP and D-dimer independently predicted disease progression at 6-months, with a combined model identifying almost a three-fold increased risk (HR = 2.9, 95% CI = 2.3–3.7). Radiotherapy was protective against progression on univariate analyses (HR = 0.62, 95% CI = 0.52–0.74, *P* < 0.001), although most patients received chemotherapy rather than RRT specifically [38]. Whilst extrapolation to RRT populations should therefore be cautious, these studies signal that clinicians should routinely measure baseline inflammatory biomarkers to risk-stratify lung cancer patients, as inflammation may guide decisions about treatment intensity and supportive care integration [39].

Malnutrition is common in patients with locally advanced and metastatic lung cancer, including those considered fit for treatment [40,41]. However, evidence linking malnutrition directly to outcomes is less developed than for the other cachexia biomarkers. Taranova et al. analysed 113 patients with limited-stage small cell lung cancer (SCLC) enrolled in a randomised clinical trial and found no significant association between PG-SGA short-form malnutrition risk and toxicity, PFS, or OS (*P* > 0.24 for all) [42]. Although methodologically robust, the limited sample size, SCLC-specific biology, reliance on a subjective screening tool, and inherent selection bias associated with clinical trial populations constrain its interpretation here.

## DISCUSSION

In this review, we demonstrate that multiple biomarkers of cancer cachexia – including weight loss, malnutrition, sarcopenia, and systemic inflammation – are associated with adverse outcomes in patients undergoing radical radiotherapy for both HNC and lung cancer. While the strength of evidence varied between cachexia domains, the overall direction of effect was remarkably consistent and aligns with the extensive literature demonstrating the prognostic importance of cachexia in patients with HNC or lung cancer treated with other modalities, particularly in the advanced setting.

An important observation is that cachexia should not be viewed simply as a consequence of advanced cancer. Although much of the published literature relates to metastatic or incurable disease, evidence suggests that cachexia can be detected and measured even in patients with potentially curable cancers. The landmark TRACERx Lung study demonstrated that patients with early-stage NSCLC undergoing surgical resection who had low skeletal muscle or adipose tissue reserves experienced significantly poorer lung cancer-specific and overall survival [43,44]. Patients with features of cachexia had distinct tumour genomic and transcriptional profiles characterised by an enrichment of inflammatory signalling pathways, and a significant association between circulating GDF15 and weight loss, skeletal, and adipose tissue at relapse was observed. Although this study was in early-stage patients undergoing surgical resection, it is perhaps the most robust link between cachexia, survival, and the molecular mechanisms behind this process.

A consistent limitation across the literature was the reliance on retrospective studies, heterogeneous patient populations, and variable definitions of cachexia-related phenotypes. This was particularly apparent for sarcopenia, where differing imaging modalities, anatomical landmarks, and diagnostic thresholds substantially limited comparisons between studies. Similar challenges were evident for inflammatory biomarkers, where numerous prognostic scores and cut-offs have been proposed without external validation. Consequently, although the evidence supports the prognostic significance of cachexia-related biomarkers, there remains uncertainty regarding which markers should be prioritised for routine clinical use.

The key clinical question now is how best to incorporate this knowledge into routine clinical practice. Nutritional assessments are embedded within standard care pathways for HNC, reflecting the recognised nutritional challenges associated with treatment. In contrast, despite similarly high levels of need, this is not the case for patients with lung cancer in the UK [41]. It is likely that dietetic assessment and appropriate intervention will be introduced to all patients with cancer in Scotland as part of a widespread prehabilitation programme. Nutrition is one of three pillars of prehabilitation, along with physical activity and psychological support [45–47]. The aim is to implement widespread nutritional screening using the PG-SGA. Intervention will then be tailored to the level of need, defined at a universal, targeted, or specialist level [48].

This screening approach will identify those who have high existing nutritional needs but does not adequately identify those with pre-cachexia who have not yet experienced clinically apparent weight loss or functional decline. Measuring early body composition changes or systemic inflammation may identify patients who could benefit from supportive intervention for cachexia before the clinical effects arise. An important challenge here is whether the four domains assessed in this review should be considered separately or whether a single robust biomarker or composite score could be implemented as a pragmatic assessment of cachexia impact in routine practice.

At present, clinicians rarely use objective cachexia biomarkers to guide treatment decisions. Factors such as weight loss, nutritional status, albumin concentration, and systemic inflammation are often considered implicitly as part of the holistic clinical assessment rather than being incorporated into formal decision-making frameworks. However, cachexia has the potential to improve prognostic accuracy, and support shared decision-making. Our group has previously suggested that the role of these biomarkers is not necessarily to determine treatment eligibility but to facilitate more informed discussions regarding expected benefits, toxicity risk, and supportive care needs [20,49]. We recently examined the probability of patients receiving adjuvant immunotherapy after chemoradiotherapy for locally advanced NSCLC. Patients with normal routine albumin and neutrophil counts are almost twice as likely to start durvalumab as those with high levels of inflammation (84% vs. 43%, *P* ≤ 0.001) [50]. Such information may help clinicians and patients better understand the likelihood of completing multimodality treatment pathways. This may ultimately support treatment intensification for those with favourable risk profiles or treatment de-escalation for those unlikely to derive a meaningful benefit.

Beyond prognostication, effective pharmacological treatment of cancer cachexia remains an unmet need, and current evidence remains insufficient to recommend anti-cachexia therapy in patients receiving RRT [51]. Encouragingly, a recent phase II randomised placebo-controlled trial of ponsegromab, a GDF-15-inhibiting monoclonal antibody, demonstrated significant dose-dependent weight gain in patients with cancer cachexia at 12-weeks, alongside improvements in appetite and physical activity [52]. Similarly, anamorelin has demonstrated beneficial effects on body weight and lean body mass in several studies and is already used routinely in some parts of the world [53,54]. Significantly, the benefit of anamorelin appears to be independent of background inflammatory status, raising the possibility that its benefits may extend to those with pre-cachexia traits [55]. Although these therapies have primarily been studied in advanced disease, they represent an intriguing future opportunity for patients undergoing radical treatment, where preserving fitness, maintaining treatment intensity, and reducing toxicity may translate into meaningful improvements in long-term survival.

## CONCLUSION

Cancer cachexia is an important but under-recognised predictor of outcomes in patients with lung or HNC receiving radical radiotherapy. Prospective validation and integration into clinical trials are needed to support personalised treatment decisions and future anti-cachexia therapeutic strategies.
